# Inflammatory bowel diseases and the low-FODMAP diet: benefits and challenges in therapy

**DOI:** 10.3389/fnut.2025.1673867

**Published:** 2025-10-08

**Authors:** Kinga Skoracka, Alicja Ewa Ratajczak-Pawłowska, Martyna Marciniak, Anna Maria Rychter, Klara Szwarc, Liliana Łykowska-Szuber, Agnieszka Dobrowolska, Iwona Krela-Kaźmierczak

**Affiliations:** ^1^Department of Gastroenterology, Dietetics and Internal Diseases, Poznan University of Medical Sciences, Poznan, Poland; ^2^Doctoral School, Poznan University of Medical Sciences, Poznan, Poland; ^3^Laboratory of Nutrigenetics, Department of Gastroenterology, Dietetics and Internal Diseases, Poznan University of Medical Sciences, Poznan, Poland

**Keywords:** Crohn’s disease, ulcerative colitis, low-FODMAP diet, prebiotics, gut microbiota, nutritional deficiencies

## Abstract

Inflammatory bowel disease (IBD) is a chronic condition characterized by periods of exacerbation and remission, during which patients experience a range of gastrointestinal symptoms that negatively impact their quality of life. With the rising prevalence of the Crohn’s disease (CD) and ulcerative colitis (UC), new therapeutic approaches, including nutritional strategies, are being sought to support therapeutic treatment. One of dietary strategy under investigation is a diet low in fermentable oligosaccharides, disaccharides, monosaccharides and polyols diet (LFD), which limits the intake of indigestible and slowly absorbed carbohydrates. An increasing number of studies indicate that the LFD may alleviate visceral hypersensitivity and improve patients’ quality of life. However, alongside these benefits, the diet carries risks, particularly in regard to potential adverse effects on the gut microbiota and the possibility of vitamin and mineral deficiencies. The current body of evidence indicates that the LFD is best considered as a short-term therapeutic option for IBD patients in remission with persistent IBS-like symptoms. While there is evidence to suggest that it is effective in controlling symptoms, there is absence of data demonstrating that it reduces intestinal inflammation. Therefore, its use requires careful management to mitigate risks related to nutritional status and gut microbiota.

## 1 Introduction

Inflammatory bowel disease (IBD), which includes ulcerative colitis (UC) and Crohn’s disease (CD), is a multifactorial disease in which environmental factors unquestionably play a role. This is confirmed by the fact that the incidence of IBD is rapidly increasing in developing countries. A new trend is also observed in highly developed countries, where the incidence is clearly increasing not only among children but also in older age groups. According to Global Burden of Disease Study 2019, there was approximately 4.9 million cases of IBD reported worldwide in 2019 (1). Among European countries, the highest incidence of UC is observed in Norway—505 per 100.000, while for CD in Germany—322 per 100.000 people. In North America, UC is most common in the USA—286 per 100.000, and CD in Canada—319 per 100.000 people. Since 1990, the incidence of IBD has been increasing significantly in newly industrialized countries of Africa, Asia and South America (2). Therefore, IBD can therefore currently be considered a global disease. The etiopathogenesis of these diseases is multifactorial. Genetically determined immune mechanisms are of pivotal significance in this regard, with these mechanisms being activated in an excessive and uncontrolled manner in response to triggers such as diet and the intestinal microbiome. The results of studies aimed at characterizing the microbiota of patients suffering from IBD indicate a generalized decrease in its biodiversity and a taxonomic reduction of *Firmicutes* and *Bacteroidetes, Lactobacillus* and *Eubacterium*. In patients with IBD, a reduction in butyrate-producing species and short-chain fatty acids (SCFAs), which are responsible for maintaining proper intestinal homeostasis and reducing inflammation, is also observed (3). Undoubtedly, dietary factors influence the stabilization of the intestinal microbiome and the production of nutritious butyrates. The fact that one of the basic environmental factors that has changed in developing countries is the increasing popularity of the Western diet confirms the thesis that the role of dietary factors in the development of IBD is indisputable. The connection between the increase in the incidence of IBD in these regions and the change in the way of eating is not accidental. Many studies indicate the connection between a high-fat diet, rich in animal protein, carbohydrates and food additives used to preserve and improve its quality, and the increased risk of developing and exacerbating IBD. They are not only responsible for changes in the microbiome, but also have an adverse effect on the integrity of the intestinal epithelium and the production of protective factors (4, 5). In last years, both clinicians and patients have shown increasing interest in diet as an easily modifiable environmental factor and a possible option for the prevention and treatment of IBD (6). Despite the fact that the incidence of IBD is increasing worldwide, the progress in the treatment of this group of diseases has resulted in the majority of patients achieving long-term remission of the disease. However, this is associated with new challenges. It is estimated that over 30% of patients with IBD present with irritable bowel syndrome (IBS) symptoms. This is related to the fact that these patients, despite achieving remission of the disease, still present a low quality of life. Based on observations of patients with IBS, it is known that the role of diet in the treatment of this disease is indisputable. Recently, much attention has been paid to the dietary restriction of fermentable oligosaccharides, disaccharides, monosaccharides and polyols (FODMAPs) due to their poor absorption in the gastrointestinal tract. In IBS, a low-FODMAP diet (LFD) has been shown to alleviate adverse gastrointestinal symptoms by decreasing bacterial fermentation in the large intestine due to a reduced osmotic load effect in the small intestine and colon, which reduces gas production and suppresses the immune response in the intestine. A diet low in products rich in FODMAPs has a well-established position in the treatment of IBS symptoms and has been recognized as a complementary therapy in its treatment (7–9). The interest in the LFD has therefore also appeared in the aspect of treating IBD. This diet is being studied both as an element that helps reduce visceral hypersensitivity accompanying these diseases and as a factor that may contribute to inducing and maintaining remission of these diseases. In this paper, we have extensively reviewed the effects of macronutrients, vitamins, minerals and prebiotics on IBD risk and course of the disease and discuss the indications and limitations of LFD use in supporting IBD treatment, to facilitate the safe navigation of the dietary therapy process.

## 2 Nutrition in IBD

Diet seems to play a role in both the occurrence and the course of IBD. Many studies focus on finding a connection between food groups or nutrients and the progression of the development of IBD (10). On the other hand, the course of the disease may affect the nutritional status of patients. In patients with IBD, malnutrition may manifest as low body weight and reduced body mass index (BMI), reflecting an imbalance in energy and nutrient intake. This can result in protein-energy malnutrition as well as deficiencies in essential micronutrients (11). It is vital to note that malnutrition is common among patients with IBD, affecting up to 60% of individuals and significantly impairing quality of life (12). Moreover, malnutrition is also diagnosed in patients in remission. Studies indicate that more than 5% of IBD patients in remission are underweight, nearly 10% are malnourished, and almost 40% are at risk of malnutrition (13). Malnutrition may result from impaired digestion, malabsorption, chronic inflammation, increased metabolic demand or inadequate dietary intake (14). Furthermore, malnutrition influences the course of the disease. Protein-energy malnutrition increases the risk of mortality and hospital readmission among patients with IBD (15).

Importantly, deficiencies of vitamins and minerals may also occur in patients without clinical signs of malnutrition, highlighting the need for a detailed discussion of their risk.

Nutritional deficiencies may not only exacerbate systemic outcomes but also exert a direct influence on the composition of the gut microbiota and result in the abundance of butyrate-producing species, thereby contributing to the gut microbiota changes characteristic for IBD, what is discussed in Section 3.

### 2.1 Fats

The impact of fat and fatty acids on the development and course of IBD is still being studied. However, it has been observed that fatty acids can exhibit both pro-inflammatory and anti-inflammatory effects and can play a regulatory role in immunity by affecting intestinal barrier permeability. For example, a high-fat diet rich in saturated fatty acids (SFA) shows pro-inflammatory effects. This is supported by a study which demonstrated that a high intake of fat, especially cholesterol and animal fat, increases the risk of UC. Additionally, a high-fat diet affects gut immunity by influencing intercellular tight junctions, mucin 2 secretion, and antimicrobial peptide production. A meta-analysis, on the other hand, shows that supplementation with polyunsaturated fatty acids (PUFA) has a weak or no effect on the development and course of IBD (16). Głąbska et al. have not find differences in total fat intake, SFA, cholesterol or PUFA between UC patients in remission and healthy volunteers (17).

However, it has been documented, according to the guidelines of the European Society for Clinical Nutrition and Metabolism (ESPEN), that a diet high in omega-3 fatty acids and low in omega-6 fatty acids reduces the risk of developing IBD (18). Moreover, the American Gastroenterology Association (AGA) recommends a Mediterranean diet, rich in fats (about 40% of energy is driven from fat), mainly monounsaturated fats, for all IBD patients (19, 20). However, the beneficial effects of the Mediterranean diet are caused by fatty acid composition and other elements of this diet, e.g., high intake of vegetables or fruits.

### 2.2 Proteins and amino acids

In a systematic review by Potcovar et al., the incidence of sarcopenia in patients with IBD ranged from 36.7 to 65% (21). Muscle mass deficiency results in prolonged hospitalization, poorer response to treatment, including biological treatment, and longer and more difficult recovery after surgery (22). In people with inflammatory bowel disease with an active form of the disease, according to the ESPEN recommendations from 2023, an increased protein intake of 1.2–1.5 g/kg/d is recommended for adults, while in the case of remission, a similar intake to the general population is recommended—1 g/kg/d (18).

Scientific studies have shown that supplementation with both whey and soy protein can increase skeletal muscle mass, hemoglobin and iron levels, creatinine levels, and albumin levels in patients with IBD (23–25). Lactoferrin, a protein isolated from milk, has anti-inflammatory and immunomodulatory effects, which can significantly reduce the levels of inflammatory markers and improve biochemical parameters—hemoglobin and iron levels in patients with non-specific IBD (26).

It is worth noting that abnormalities in amino acid metabolism have been observed among patients with IBD. Glutamine levels in blood and colon of IBD patients in both remission and active disease are reduced compared with controls (27). Parenteral glutamine is thought to have anti-inflammatory effects, which may potentially improve clinical outcomes in Crohn’s disease (28). However, according to Kohei et al., enteral nutrition with added glutamine or total parenteral nutrition did not show benefit in children with CD and adults with CD and UC (27). In the case of arginine, changes in metabolism have been observed in patients with inflammatory bowel disease, and its supplementation ameliorated experimental colitis. Coburn et al. showed that arginine levels are decreased in patients with UC and its levels correlate with disease severity. Changes in histidine, glycine, and threonine levels have also been observed in serum and tissues of patients with IBD (29).

### 2.3 Carbohydrates

In a systematic review conducted by Hou et al. in 2011, the authors focused on the type of food consumed and the risk of IBD, finding that high intake of fruit and dietary fiber correlated with a low risk of developing IBD. This is due to the production of SCFAs and lactate, which inhibit the growth of potentially pathogenic organisms, a shortened intestinal transit time that limits bacterial adhesion to the intestinal wall, and reduced production of harmful substances that may contribute to the IBD pathogenesis (30). Scientific data also describe Specific Carbohydrates (SCD) which emphasizes simple sugars—glucose, galactose, fructose along with fresh vegetables, fruits, unprocessed meat, nuts, yogurts and hard cheeses, while excluding dairy products and complex carbohydrates found in cereals (31). One of the largest studies on SCD compared it with the Mediterranean diet. The randomized study included 194 patients, the protocol lasted 6 weeks; no differences were observed between the groups in terms of clinical response, calprotectin, or C-reactive protein (CRP), although more than 40% of patients achieved remission (32).

According to the International Scientific Association for Probiotics and Prebiotics, prebiotics are defined as “a substrate that is selectively fermented by intestinal microflora and confers a health benefit to the host” (23). This group includes, e.g., inulin, lactulose, lactosucrose, fructoligosaccharides (FOS) and galactooligosaccharides (GOS) (24). Studies illustrate their mixed effects: Benjamin et al. administered FOS to patients with active CD for 4 weeks and observed a worsening of the clinical condition of CD and no significant differences in the levels of *Bifidobacteria* spp. and *F. prausnitzii* compared with controls (33). This may be explained by excess fiber that can exacerbate intestinal symptoms through mucosal irritation and increased fermentation. Conversely, Casellas et al. provided FOS and inulin to individuals with active UC and found a decrease in calprotectin after just 1 week (25).

This beneficial role of prebiotics and SCFA production also highlights a key paradox of the low-FODMAP diet: while the restriction of these substrates may reduce symptoms, it simultaneously limits the very mechanisms that support gut homeostasis.

### 2.4 Micronutrients

Beyond the general problem of malnutrition, patients with IBD have an increased risk of nutritional deficiencies, including vitamins, such as vitamin D, folic acid, vitamin B12 and minerals, with most common iron deficiency (14). This is mainly related to diarrhea, poor absorption, intestinal failure, and anorexia associated with active disease (11). According to the 2023 ESPEN guidelines, it is necessary to monitor micronutrient levels at least once a year (18).

In the case of vitamin D, ESPEN experts suggest monitoring its concentration in both adults and children with an active form of the disease, treated with corticosteroids, and suspected 25(OH) vitamin D deficiency; if the result indicates deficiency, it is recommended to include vitamin D/calcium supplementation (18). Factors influencing insufficient vitamin D concentration include male gender, Asian origin, Crohn’s disease, surgery related to IBD, use of steroids, use of biologics, low body mass index and avoidance of dairy products (18, 34). Increasingly, scientific data indicate that vitamin D may be an immunomodulatory factor that may affect the course of the disease in patients with nonspecific inflammatory bowel diseases (35). A correlation is also noted between low vitamin D concentration and the course, activity of the disease and the occurrence of another exacerbation (36, 37). Vitamin D deficiency correlates with a higher risk of osteopenia and osteoporosis (33).

One of the more serious deficiencies is anemia, which occurs in 60–80% of patients with Crohn’s disease. In the case of patients with UC, the occurrence of anemia (66%) is due to iron deficiency, which constitutes a significant majority compared to patients in the CD group (39%). The causes of anemia can include limited meat consumption, chronic blood loss leaking from the altered mucosa of the intestine and absorption disorders. Iron administered orally and intravenously is an effective form of treating anemia in IBD, however, according to Gkikas et al., oral iron supplementation can increase dysbiosis and intestinal inflammation, therefore high doses of oral iron should be avoided and a low initial dose such as one tablet of ferrous sulfate daily should be considered (38, 39).

Furthermore, in patients with nonspecific IBD, common problem is also folic acid deficiency caused by low intake (exclusion/consumption of small amounts of fresh vegetables and fruits; decreased/loss of appetite associated with gastrointestinal symptoms), impaired absorption, as well as the use of sulfasalazine and methotrexate (18, 40, 41). Scientific studies have noted that patients with CD are particularly susceptible to folate deficiency—the location of the disease and the use of sulfasalazine (14). The most common consequence of vitamin B9 deficiency is megaloblastic anemia, however, some studies also note low bone mineral density and hyperhomocysteinemia (40). The 2020 European Crohn’s and Colitis Organization (ECCO)-ESPGHAN guidelines suggest that patients taking methotrexate should receive 5 mg of folate once a week (24–72 h after methotrexate administration) or 1 mg daily for 5 days a week—these data apply to both adults and children (42). Moreover, due to the fact that the absorption of vitamin B12 occurs mainly in the distal part of the small intestine, according to the ESPEN guidelines, patients with CD who have undergone resection of more than 20 cm of the small intestine should receive vitamin B12 supplementation (32, 38). In the case of documented clinical deficiencies, it is recommended to administer 1,000 μg of vitamin B12 (intramuscular injection) every other day for a week, and then monthly for the rest of life, while in patients who have had an ileum resection > 20 cm, 1,000 μg of vitamin B12 should be administered prophylactically also monthly and indefinitely (18).

It is also worth noting that almost half of patients with IBD struggle with zinc deficiency (38.5%). The problem mainly concerns patients with CD, also those who show a higher daily zinc intake compared to the control group (65%). Increased zinc losses are closely related to the type, weight of stool and malabsorption syndromes (39, 43).

Studies also show reduced selenium concentration by up to 35–40% (38) in adult patients with CD in both active and inactive forms of the disease. However, in studies conducted on children, the results do not support the above hypothesis (43).

Given the inflammatory nature of IBD, there is a need for consideration of antioxidant vitamins. Reduced levels of vitamin A, beta-carotene, and vitamin C have been observed among patients. Importantly, serum beta-carotene levels were found to be reduced in patients with both active and inactive CD, and reduced vitamin E levels were observed in patients with UC, regardless of disease severity (44).

There are also studies indicating significant vitamin E deficiency in patients with CD compared to controls. Among adolescents with CD, vitamin E deficiency is the most common antioxidant vitamin deficiency (45). It was observed that patients struggling with the active form of CD after the introduction of vitamin E and vitamin C supplementation showed less oxidative stress compared to the control group (46).

### 2.5 Macronutrients

About 13% of adults with CD and 10% of patients with UC are calcium deficient, which occurs especially in the early stages of the disease. Additionally, inadequate dietary calcium intake has been reported in 70% of premenopausal women with IBD (47). Calcium deficiency results in bone loss and increases the risk of osteoporosis. Important causes that may affect too low blood calcium levels include vitamin D deficiency and the use of restrictive diets excluding milk and dairy products, which are often used by patients with IBD. In cases where patients are unable to maintain an adequate calcium intake of 1.5 g/day, supplementation of 500–1,000 mg daily should be considered (38). In a study by McCarthy et al., vitamin D and calcium supplementation among patients with IBD was found to have a positive effect on bone health (48). However, it is worth noting that the use of sodium fluoride or ibandronate as an adjunct to calcium and cholecalciferol supplementation among patients with CD did not demonstrate additional benefits with respect to bone mineral density including fracture prevention (49). Research also points out that patients with newly diagnosed UC compared to the control group struggled with phosphorus deficiency, which is essential for the proper functioning of the body (50).

Moreover, approximately 6–20% of patients with CD present with potassium deficiency which may influence the course of the disease (38). According to Reif et al. increased intake of potassium, magnesium, and water may reduce the risk of inflammatory bowel disease, especially Crohn’s disease (51). Among patients with IBD, the daily potassium intake was significantly reduced and was 707.30 mg for men and 714.47 mg for women, respectively (52).

Patients with IBD also show reduced daily sodium intake compared to the control group. According to Yin et al., male subjects consumed 125.63 mg sodium and female subjects 141.57 mg sodium per day, respectively, which significantly deviates from the recommended reference nutrient intake (RDI) (52).

Similarly about 14–33% of CD patients are magnesium deficient (47). This deficiency may result from inadequate dietary intake, hypocalcaemia, increased intestinal loss, malabsorption syndrome, bacterial overgrowth, or small bowel resection.

### 2.6 Other antioxidants

Furthermore, increased production of reactive oxygen species and decreased antioxidant protection in damaged tissues have been observed in IBD (43). Daily consumption of anti-inflammatory anthocyanin-rich blueberry juice over 6 weeks has been shown to induce remission in 63.7% of patients with mild UC and 90.9% of patients with moderate UC (46). The presence of flavones and resveratrol was also associated with a reduced risk of CD (39). In contrast, a study of oral curcumin supplementation observed that curcumin at a dose of 3 g per day compared with placebo showed clinical remission in non-severe UC (46).

### 2.7 Food additives

A highly processed diet correlates with increased consumption of food additives, including emulsifiers. It has been observed that increased intake of emulsifiers is associated with the pathogenesis of IBD through abnormal host-microbiome interaction, which may cause intestinal inflammation and colon carcinogenesis. Artificial sweeteners and phosphates are also included in food additives and may induce intestinal dysbiosis. In a study conducted in CD mice, artificial sweetener consumption increased the risk of *Proteobacteria* development and increased bacterial influx into the ileal lamina propria. It has been observed that consumption of foods rich in food additives, such as processed meats and beverages, increased the risk of developing or recurring IBD (27). Food additive-related components, including titanium dioxide and aluminosilicate microparticles, exacerbate inflammation in Crohn’s disease by accumulating in intestinal lymphoid tissue macrophages. However, the results of other researchers on this topic are equivocal (47).

## 3 Microbiota disorders in IBD

The gut microbiota plays a crucial role in maintaining intestinal health. However, defining what constitutes a ‘healthy’ microbiota still poses a challenge due to its complexity and multiple influencing factors, including diet, medications, and lifestyle (53, 54). While markers such as microbial diversity and the *Firmicutes/Bacteroidetes* (F/B) ratio are frequently the focus of study, it is the functional capabilities such as metabolite production and immune regulation which may offer more informative insights with regard to health outcomes (55).

Research on the microbiome in IBD is complicated by the influence of therapeutic interventions and the impact of ongoing inflammation. Additionally, much of the existing evidence comes from cross-sectional analyses rather than prospective longitudinal cohort studies. Despite these challenges, the gut microbiota remains a key focus of IBD research, as its composition and function are increasingly recognized as critical contributors to disease pathogenesis, progression, and potential therapeutic strategies (56).

Several studies have investigated the role of the F/B ratio in the occurrence and progression of UC (57). For example, Tsai et al. a decrease in *Firmicutes* and an increase in *Bacteroidetes* during active disease in both CD and UC (58). After biologic treatment, these trends reversed, suggesting a possible correlation between the F/B ratio and treatment efficacy. Furthermore, alpha diversity was significantly reduced in active CD patients but gradually increased following biologic therapy, paralleling clinical improvement (58). In another study, *F. prausnitzii* levels were significantly lower in patients with UC and their relatives compared to controls (patients median: 1.4 × 10^8^ vs. controls 6.5 × 10^8^, *p* < 0.0001). Low *F. prausnitzii* counts correlated with shorter remission (<12 months) and higher relapse rates (>1 relapse/year; *p* < 0.01). During follow-up, patients maintaining remission showed a gradual increase in *F. prausnitzii* to levels comparable with controls, while those who relapsed had persistently low levels (*p* < 0.05) (59). Notably, *F. prausnitzii* abundance were shown to be negatively correlated with IBD activity in several meta-analyses; however, cut-off concentrations in diagnosing or treating IBD are not yet established (60–62).

In CD, the abundance of bifidobacteria in the mucosal microbiome has been shown to be positively associated with the proportion of IL-10–expressing dendritic cells. Converserly, IL-6 production and TLR-4 expression have been demonstrated to correlate negatively with *F. prausnitzii* (63).

This baseline altered microbiota composition is particularly relevant when considering dietary interventions such as the LFD, which serves to further modulate the availability of fermentable substrates for gut microbes.

The treatment itself has also been demonstrated to affect the gut microbiota. In the study of Morgan et al., use of mesalamine (5-aminosalicylic acid) was associated with a marked decrease in *Escherichia/Shigella* abundance by over 100% relative to the average (*q* < 0.04) (64). Additionally, both mesalamine and immunosuppressants led to modest increases in *Enterococcus*, with immunosuppressant-treated patients showing a statistically significant rise exceeding 100% of average abundance (*q* < 0.09). Given these interactions between treatment and the microbiota, it is reasonable to explore adjunctive therapies such as probiotics to modulate microbial balance. Indeed, a separate subgroup analysis in the study of Estevinho et al., demonstrated that combining 5-ASA with probiotics may improve remission rates in mild-to-moderate ulcerative colitis (OR 2.35, 95% CI 1.29–4.28) (65). Probiotics also significantly lowered relapse risk in recurrent pouchitis (OR 0.03, 95% CI 0.00–0.25) and showed a trend toward reducing clinical recurrence in inactive ulcerative colitis (OR 0.65, 95% CI 0.42–1.01), although no protective effect was found for Crohn’s disease. In this study, multi-strain probiotic formulations appeared more effective in inducing remission and preventing relapse in ulcerative colitis, yet probiotic use was not linked to improved endoscopic outcomes. It should be also remembered that in the American College of Gastroenterology (ACG) guidelines indicate that probiotics may have potential as an adjunctive treatment for ulcerative colitis, but there is insufficient evidence to recommend their routine use and each case should be considered individually.

In some IBD patients, intestinal surgery is unavoidable as part of disease management. Analysis of metagenomic and metabolomic data from an IBD cohort indicates that such surgeries can have lasting impacts on the gut microbiome, leading to decreased microbial and metabolic diversity and heightened instability within the gut ecosystem. In the study of Fang et al., surgery had a decreasing effect on the alpha diversity. However, although the changes in microbiome were observed, metabolome seemed to remain unchanged. Importantly, the type of surgery accounted for the largest proportion of variation in microbiome composition (9.84%), surpassing other factors such as disease subtype, antibiotic use, and disease activity (66).

In summary, although therapeutic interventions and surgeries significantly influence gut microbiota composition and diversity in IBD, the microbiome remains central to understanding disease dynamics and guiding treatment. Continued longitudinal studies integrating microbial, metabolic, and clinical data are essential to unravel the complex interactions driving IBD pathogenesis and therapy response.

## 4 Low-FODMAP diet

### 4.1 LFD—definition

FODMAPs represent a group of short-chain carbohydrates characterized by poor absorption, rapid fermentation, and high osmotic pressure (Table 1). These properties result in the accumulation of water and gas, leading to distension and visceral hypersensitivity. Consequently, this can exacerbate gastrointestinal symptoms (67, 68).

**Table 1:** Sources of FODMAPs in the diet (70).

FODMAP group	Example sources	Explanation of mechanism
Oligosaccharides—fructans and galacto-oligosaccharides (GOS)	Wheat, rice, legumes, nuts, artichokes, onions, garlic	Fructans: chains of fructose molecules GOS: undigested in large colon forms of carbohydrates
Disaccharides—lactose	Milk and milk products	Deficiency of lactase enzyme
Monosaccharides—fructose	Apples, pears, watermelon, mango, honey, sugar snap peas, corn syrup	Lack of balance between glucose and fructose ➔ poorly absorption of fructose
Polyols—sugar alcohols	Apples, pears, cauliflower, mushrooms, stone fruit, sugar snap peas, sugar-free chewing gum, sugar-free sweets	Poorly absorbed sugar alcohols

The acronym was first used in the literature in the early 20th century by Gibson and Shepherd of the University of Monash to denote the concept that excessive dietary provision of FODMAPs could be a dietary factor underlying susceptibility to developing CD (69). Subsequent years of intensive research have led to the development of the LFD, now considered an effective management strategy for IBS. It is estimated that in 50–84% of patients with IBS, dietary factors, particularly FODMAPs, play a key role in the development of symptoms (70). Nevertheless, while the application of LFD is predominantly ascribed to the management of IBS, there has been a resurgence of interest in employing this nutritional model as a support for IBD therapy (71).

LFD is a temporary dietary intervention intended for short-term use. It comprises three phases: elimination, reintroduction, and personalization of the diet. The first stage, which is characterized by a significant reduction in FODMAPs intake to a level that does not cause symptoms, lasts 4–8 weeks. The subsequent reintroduction stage, spanning 6–10 weeks, entails the gradual reintroduction of single FODMAPs-containing products into the diet, in accordance with a structured testing protocol designed to ascertain patient tolerance to the products that were excluded during the initial elimination phase. The final phase, the personalization phase, involves adjusting the diet to align with the patient’s individual requirements, while minimizing the number of product groups, as determined by observations made during the second phase of the diet (70).

### 4.2 Application of LFD in IBD

In recent years, researchers have conducted a number of studies to evaluate the effectiveness of LFD in patients with IBD and comorbid symptoms such as bloating, abdominal pain, diarrhea and frequent bowel movements (9). In 2024, the AGA published a Clinical Practice Update in which, based on existing scientific evidence, it notes that the LFD diet may be worth trying in IBD patients with IBS-like symptoms (72). It appears that IBD patients are more likely to experience IBS-like symptoms than the healthy population.

Halpin et al. conducted a meta-analysis and systematic review including 13 studies. They noted that almost 40% of IBD patients had IBS-specific symptoms. Moreover, IBS-typical symptoms were also significantly more frequent in patients with CD than in those with UC—46% vs. 36%, and also among those with active disease. Nevertheless, almost a third of IBD remission also reported IBS-typical symptoms (73). Furthermore, 8 years later, Faibrass et al. conducted a meta-analysis and systematic review to assess the frequency of IBS symptoms in IBD patients in remission. Among 3,169 patients in remission, 32.5% had symptoms typical of IBS. Again, symptoms were more common in patients with CD than UC—36.6% vs. 28.7% (74).

It is debatable whether the IBS symptoms observed in IBD patients in remission are indicative of co-morbid IBS are a manifestation of IBD with persistent subclinical inflammation. However, it should be emphasized that the Rome criteria for IBS clearly state that IBS can be diagnosed when all organic causes of symptoms, including IBD, are excluded (75).

Referring to the efficacy of the LFD in patients with IBD, it is worth citing the study by Cox et al. (76) who conducted a randomized, controlled trial in a group of 52 patients with IBD in remission but with persistent intestinal symptoms, to investigate the effect of a 4-week intervention using the LFD on patients’ persistent intestinal symptoms, gut microbiome and circulating inflammatory markers. The researchers observed that 52% of those in the low LFD group reported an improvement in the severity of intestinal complaints compared to 16% in the control diet group. What is important, patients on the low-FODMAP diet exhibited significantly lower abundance of *B. adolescentis*, *B. longum*, and *F. prausnitzii* compared to those on control diet, which could have implications for the mucosal immune response and disease course, given the immune-regulatory functions of these bacteria, as previously discussed. It is important to note that these alterations coincide with the characteristic dysbiosis observed in IBD, including a reduction in the number of butyrate-producing species, as outlined in Section 3. Thus, although the LFD has been demonstrated to alleviate IBS-like symptoms, it may also exacerbate microbial imbalances that are considered to be a contributing factor to the pathogenesis of IBD (76). It can be hypothesized that these microbial alterations are a consequence of changes in colonic fermentable substrate. Bifidobacteria have been shown to preferentially ferment fructans and GOS, while *F. prausnitzii* uses them indirectly through cross-feeding (77). Moreover, in discussed study by Cox et al. patients observed an improvement in health-related quality of life, but no significant differences in microbiome diversity, clinical disease activity or inflammatory markers were observed (76).

The mechanism of action of a LFD in patients with IBD in remission with concomitant gastric complaints is thought to mirror that described in IBS, where reducing osmotic load in the small intestine and gas production alleviate symptoms as bloating, abdominal pain and diarrhea (78).

Table 2 shows the results of the randomized trial on the effect of a FODMAPs-restricted diet on IBD. Numerous non-randomized studies have also been conducted, in which positive results have been observed concerning the use of the LFD (78–82).

**Table 2:** Summary of randomized controlled trials on the low-FODMAP diet in IBD.

Research study	Methodology	Effect on GI symptoms	Effect on inflammatory markers	Effect on microbiome	Effect on quality of life
Halmos EP et al. 2016 (85)	9 patients with CD—1-wk LFD vs. typical Australian diet—cross-over study	bloating, pain and gas have decreased improved stool consistency	no significant change in calprotectin level	no effect on SCFA no effect on microbial abundance decrease in butyrate producing bacteria, increase of *R. torques*	not directly assessed
Cox SR et al. 2020 (76)	52 patients with quiescent CD or UC—4-wk LFD vs. control diet—single-blind trial	relief of bowel symptoms	no significant effect on markers of inflammation in the blood	no significant impact on microbiome diversity, lower presence of selected bacteria—lower abundance of *B. adolescentis*, *B.longum*, and *F.prausnitzii* vs. patients on control diet	increase in scores of health-related quality of life
Bodini G et al. 2019 (86)	55 patients with IBD in remission or with mild disease activity—6-wk LFD vs. standard diet	symptom improvement	an amelioration of fecal inflammatory markers		positive impact on quality of life even in patients with mainly quiescent disease
Pedersen N et al. 2017 (8)	89 patients with IBD in remission or with mild-to-moderate disease and IBS—6-wk LFD vs. normal diet—open-label trial	reduced IBS-like symptoms	not reported	not reported	increased quality of life in patients with IBD in remission

CD, Crohn’s disease; GI, gastrointestinal; IBD, inflammatory bowel diseases; LFD, low-FODMAPs diet; UC, colitis ulcerosa.

Despite the benefits that the LFD appears to provide for IBD patients with persistent gastrointestinal symptoms, it should be noted that many products excluded or limited in the first phase of the LFD have prebiotic actions that normally support the growth of beneficial gut bacteria including butyrate-producers species (83). Restricting these substrates may reduce the abundance of bifidobacteria and *F. prausnitzii* and lower SCFA production, a mechanism particularly relevant in IBD where such deficits are already present. SCFAs influence gene expression in cells, thus contributing to regeneration and reducing intestinal inflammation. They also lower intestinal pH, which may limit the growth of pathogenic bacteria. Although some studies report a beneficial modulation of the gut microbiota in subsequent phases of the LFD—manifested by an increase in *F. prausnitzii* and *Firmicutes* and a reduction in *Bacteroidetes—*the long-term impact of such shifts remains uncertain (72, 82).

In addition to microbiota-related effects, the elimination of certain dietary components increases the risk of micro- and macronutrients deficiencies, especially in patients already susceptible to malnutrition. The diet itself poses a number of practical challenges related to adherence, high costs, and difficulties in daily life (84).

For these reasons, major societies such as the AGA underline that LFD should be used as a short-term approach, followed by structured reintroduction and subsequent return to a Mediterranean-style diet. The severity of many of these difficulties can be minimized by the patient’s collaboration with a qualified and experienced dietitian with periodic follow-ups (84). To sum up, Table 3 presents future directions and clinical implications for LFD use in the course of IBD.

**Table 3:** Future directions and clinical implications for low-FODMAP diet use in the course of IBD.

Domain	Recommendation
Indications	IBD patients in remission with persistent IBS-like symptoms
Benefits	Reduction of bloating, pain, improved stool regularity, increased quality of life
Risks	Risk of malnutrition and nutrients deficiencies, reduced intake of prebiotics and SCFA production
Implementation	Short-term elimination phase ➔ structured reintroduction ➔ personalization or a simplified low-FODMAP diet model that reduces the consumption of only poorly tolerated high-FODMAP products cooperation with a clinical dietitian strongly recommended regular assessment of the patient’s nutritional status
Limitations	Limited or absent effect on inflammation and long-term disease course
Contraindications	Active flaring IBD, severe malnutrition, eating disorders, important other eliminations in the diet
Challenges	High cost, the need to cooperate with a dietitian, monotony of the diet, difficulty in following the diet
Future directions	Identification of clinical or microbial markers than can predict which patients are most likely to take advantage of this approach

## 5 Conclusion

An increasing number of studies indicate the positive impact of the LFD on the progression of IBD, particularly with regard to alleviating bloating, pain and gas, and improving stool consistency, thereby enhancing the quality of life of patients. However, the risks associated with the long-term use of a LFD—reduction of certain types of bacteria and SCFA, low diversity of diet and possible difficulties with proper diet balancing—should not be overlooked. It is therefore important to emphasize that LFD should only be introduced temporarily, especially in patients with IBS-like symptoms. To minimize the risk of negative outcomes, it is recommended that patients follow the diet under the guidance of an experienced clinical dietitian. Ultimately, it is necessary to return to a healthy, balanced Mediterranean-style diet—rich in vegetables and fruits rich in vitamins, prebiotics and polyphenols, whole-grains rich in fiber, fermented dairy rich in probiotics strains and unsaturated fatty acids in fish and high quality vegetable oils—to ensure an adequate supply of all vitamins, minerals and macronutrients, bearing in mind the greater predisposition of IBD patients to certain deficiencies and malnutrition. Further studies, that explore potential long-term effects of LFD on IBD course are desired to fully support this approach in IBD dietary treatment possibilities.

In conclusion, the evidence suggests that the overlap between microbial alterations characteristic of IBD and those induced by the LFD provides compelling evidence for the restriction of its use to short-term symptom management with the supervision of dietitian experienced in this field.
